# A pH-Sensitive Polymeric Micellar System Based on Chitosan Derivative for Efficient Delivery of Paclitaxel

**DOI:** 10.3390/ijms22136659

**Published:** 2021-06-22

**Authors:** Yang Han, Jieyi Pan, Na Liang, Xianfeng Gong, Shaoping Sun

**Affiliations:** 1 Department of Pharmaceutical Engineering, College of Chemistry and Chemical Engineering, Harbin Normal University, Harbin 150025, China; hanyang000626@163.com (Y.H.); panjieyi626@163.com (J.P.); 2 Department of Pharmaceutical Engineering, School of Chemistry and Material Science, Heilongjiang University, Harbin 150080, China; xianfenggonghlj@163.com

**Keywords:** chitosan, drug delivery, paclitaxel, pH-sensitive, polymeric micelles

## Abstract

In this study, an amphiphilic conjugate based on mPEG and cholesterol-modified chitosan with hydrazone bonds in the molecules (mPEG-CS-Hz-CH) was successfully synthesized. Using the polymer as the carrier, the paclitaxel (PTX)-loaded mPEG-CS-Hz-CH micelles were prepared by an ultrasonic probe method. The mean particle size and zeta potential of the optimized PTX-loaded micelles were 146 ± 4 nm and +21.7 ± 0.7 mV, respectively. An in vitro drug release study indicated that the PTX-loaded mPEG-CS-Hz-CH micelles were stable under normal physiological conditions (pH 7.4), whereas rapid drug release was observed in the simulated tumor intracellular microenvironment (pH 5.0). An in vitro cytotoxicity study demonstrated the non-toxicity of the polymer itself, and the PTX-loaded micelles exhibited superior cytotoxicity and significant selectivity on tumor cells. An in vivo antitumor efficacy study further confirmed that the PTX-loaded micelles could improve the therapeutic efficacy of PTX and reduce the side effects. All these results suggested that the mPEG-CS-Hz-CH micelles might be promising pH-sensitive nanocarriers for PTX delivery.

## 1. Introduction

Cancer is one of the main causes of death among human beings, and it is difficult to treat. Paclitaxel (PTX), as an effective chemotherapeutic agent, is widely used for the treatment of various cancers, such as breast cancer, ovarian cancer, lung cancer and pancreatic cancer [1]. However, there are still several barriers limiting its application—for instance, the low solubility in aqueous solution, low concentration in the target site and toxicity to normal tissue [2].

To solve these problems, various nano drug delivery systems have been designed and prepared—for example, liposomes, micelles and nanoparticles [3]. Among them, nano-scaled polymeric micelles with a core-shell structure have attracted much attention in recent years owing to their excellent biocompatibility, outstanding solubilization of hydrophobic drugs and preferential accumulation within the tumor via the enhanced permeability and retention (EPR) effect [4]. In particular, stimulus-responsive polymeric micelles are considered promising drug delivery systems for their site-specific drug release in the tumor [5].

As is widely known, there is a significant pH difference between the normal physiological condition (pH 7.4) and the tumor extracellular environment (pH 6.5–7.2), which is due to the high rate of glycolysis in cancer cells [6,7]. Moreover, much lower pH values are found at the subcellular level (pH 5.0–6.5 in late endosomes and pH 4.0–5.0 in lysosomes) [8]. Materials with pH-responsive bonds have been developed as potential carriers for the delivery of chemotherapy drugs, so as to enhance the therapeutic efficacy of the drug and decrease the side effects [9]. Hydrazone bonds have attracted much attention for their acid-labile property, meaning that they are stable at normal physiological conditions but readily cleavable in a weakly acidic tumor microenvironment [10]. It is reasonable to introduce hydrazone linkages to amphiphilic copolymers, so as to fabricate pH-sensitive polymeric micelles for the site-specific controlled release of anticancer drugs.

Chitosan (CS), a copolymer of glucosamine and *N*-acetylglucosamine, is considered an excellent biomaterial due to its outstanding properties, such as enzymatic biodegradability, non-toxicity and high biocompatibility [11]. In addition, it can easily be chemically modified due to the reactive primary amino groups and hydroxyl groups on its glucosamine units. Over the last decade, various polymeric micelles based on amphiphilic chitosan derivatives have been developed for the delivery of anticancer agents [12].

As an indispensable component of the cell membrane, cholesterol (CH) has no toxicity or immunogenicity [13]. Its high hydrophobicity due to its sterol skeleton makes CH an excellent candidate for use as the hydrophobic segment of an amphiphilic copolymer [14]. Moreover, cholesterol is a potential tumor-targeting agent, as tumor cells consume a large amount of cholesterol and overexpress the corresponding receptors [15].

Since nanoparticles are easily cleared by the mononuclear phagocyte system (MPS) before reaching the target site, it is necessary to protect the particles from rapid clearance. Surface modification with poly(ethylene glycol) methyl ether (mPEG) is a suitable strategy to achieve this [16]. mPEG is a hydrophilic, non-toxic and non-immunogenic polymer. It has been reported that mPEG coatings can shield particles from opsonization and phagocytosis [17]. It is worth noting that nanoparticles modified by mPEG display extended circulation time and improved accumulation in tumor tissues [18].

In line with the above, in the present study, an amphiphilic chitosan derivative modified with mPEG, cholesterol and hydrazone bonds (mPEG-CS-Hz-CH) was synthesized for the first time and used as a micellar system for PTX delivery. Compared with those developed in previous studies, this polymer is non-toxic and biocompatible [19]. With respect to the hydrophilicity of chitosan, the introduction of hydrophobic cholesterol could endow the copolymer with amphiphilicity to form micelles, so as to solubilize the PTX in the hydrophobic core. Moreover, cholesterol might facilitate the accumulation of PTX in the tumor. The mPEGylation ensures that the micelles have good stability in the bloodstream. In contrast, when arriving at the tumor, under the action of hydrazone bonds, the micelles can release the drug rapidly. The site-specific drug release in the tumor might enhance the antitumor effect of the drug and reduce the systemic toxicity. The synthesis of the copolymer, the fabrication and characterization of the PTX-loaded micelles as well as the in vitro and in vivo evaluations are elaborated in detail in this paper.

## 2. Results and Discussion

### 2.1. Synthesis of mPEG-CS-Hz-CH

**Figure 1:**
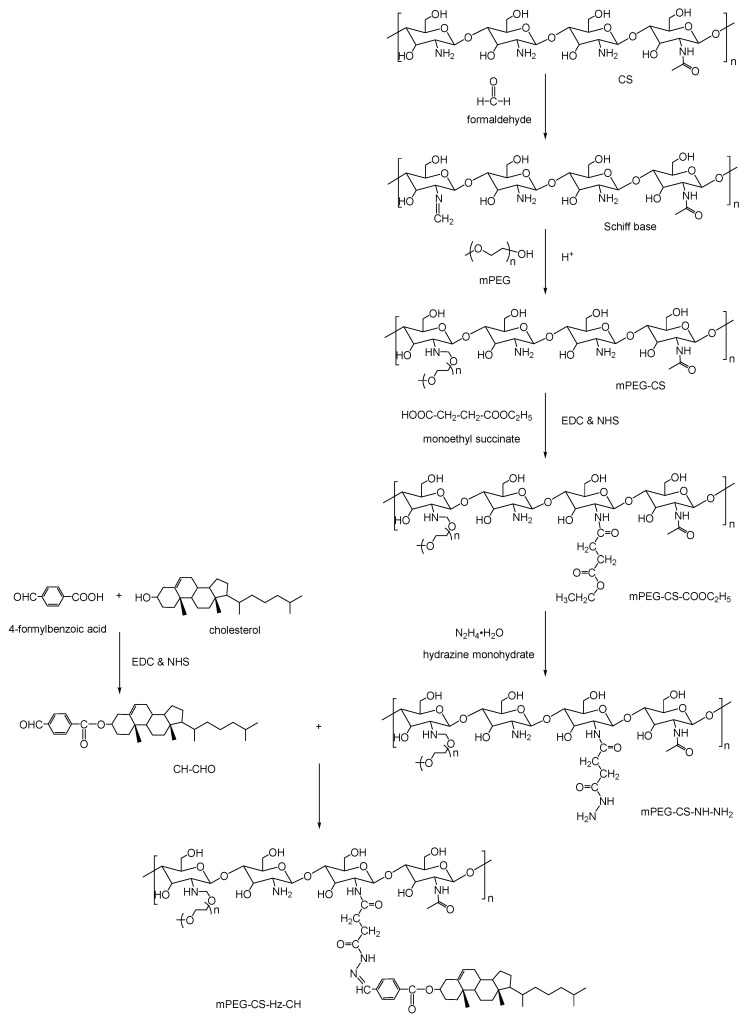
Synthesis route of mPEG-CS-Hz-CH.

Several steps were involved in the synthesis process of mPEG-CS-Hz-CH. As shown in Figure 1, CH-CHO was synthesized by attaching the carboxyl groups of 4-formylbenzoic acid to the hydroxyl groups of cholesterol in the presence of EDC and NHS. The mPEG-CS-NH-NH_2_ was prepared as follows: first, mPEG-CS was prepared by a coupling reaction between CS and mPEG. The primary amino groups of CS were reacted with the aldehyde groups of formaldehyde to obtain a Schiff base intermediate. In a slightly acidic environment, the carbon–nitrogen double bonds in the intermediate were able to further react with the hydroxyl groups of mPEG to from mPEG-CS. Next, monoethyl succinate was introduced to prepare mPEG-CS-COOC_2_H_5_ through the amide bond formation between the primary amino groups of mPEG-CS and carboxyl groups of monoethyl succinate. The reaction was performed in the presence of a carbodiimide crosslinker, which was used to activate the carboxyl groups. Then, with the effect of hydrazine monohydrate, the ester bonds in mPEG-CS-COOC_2_H_5_ were broken up, and the mPEG-CS-NH-NH_2_ formed. Lastly, the mPEG-CS-Hz-CH was synthesized by linking the acylhydrazide groups in mPEG-CS-NH-NH_2_ to the aldehyde groups of CH-CHO.

### 2.2. Characterization of mPEG-CS-Hz-CH

#### 2.2.1. Fourier-Transform Infrared (FT-IR) Analysis

**Figure 2:**
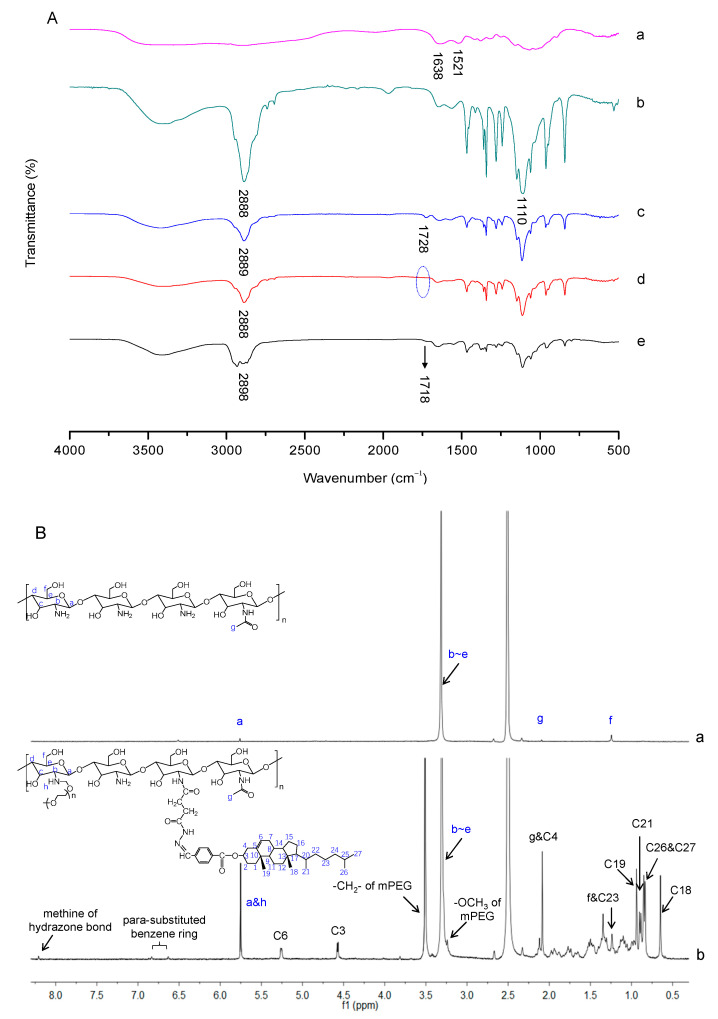
Characterization of mPEG-CS-Hz-CH. (**A**) FT-IR spectra of (**a**) CS, (**b**) mPEG-CS, (**c**) mPEG-CS-COOC_2_H_5_, (**d**) mPEG-CS-NH-NH_2_ and (**e**) mPEG-CS-Hz-CH. (**B**) ^1^H NMR spectra of (**a**) CS and (**b**) mPEG-CS-Hz-CH.

The successful synthesis of mPEG-CS-Hz-CH was verified by FT-IR analysis. As shown in Figure 2A, the FT-IR spectrum of CS showed characteristic peaks at 1638 cm^−1^ and 1521 cm^−1^, which were assigned to the C=O stretching vibration (amide I band) and N-H bending vibration (amide II band) of the amide link, respectively. For mPEG-CS, the new absorption peaks at 2888 cm^−1^ and 1110 cm^−1^ were ascribed to the -CH_2_- stretching vibration and C-O-C stretching vibration of mPEG, respectively [20]. For curve c, compared with mPEG-CS, the peak corresponding to the C=O stretching vibration of the ester bonds at 1728 cm^−1^ confirmed the successful formation of mPEG-CS-COOC_2_H_5_. In the spectrum of mPEG-CS-NH-NH_2_, the signal of ester bonds at 1728 cm^−1^ disappeared completely, which was important evidence of the synthesis of mPEG-CS-NH-NH_2_. As for mPEG-CS-Hz-CH, the typical peak of C=O stretching vibration at 1718 cm^−1^ was attributed to the ester bonds from CH-CHO, which indicated the introduction of CH-CHO. More importantly, the characteristic absorption peak of the hydrazone bond corresponding to the stretching vibration of C=N was observed at 1601 cm^−1^ [21]. All these differences indicated the formation of mPEG-CS-Hz-CH.

#### 2.2.2. Proton Nuclear Magnetic Resonance (^1^H NMR) Analysis

In order to further confirm the polymer formation, the ^1^H NMR spectra of CS and mPEG-CS-Hz-CH are presented in Figure 2B. For CS, signals at 5.75 ppm and 3.32 ppm were attributed to H (a) and H (b–e) of the saccharide units, respectively. Peaks at 2.10 ppm and 1.25 ppm were due to the protons of *N*-acetyl glucosamine and the hydroxymethyl groups, respectively [22]. Compared with CS, several new signals were observed in the spectrum of mPEG-CS-Hz-CH. The typical signals at 3.51 ppm and 3.25 ppm were due to the methylene hydrogens (-CH_2_-) and methoxyl hydrogens (-OCH_3_) of the mPEG segment, respectively. Moreover, the typical signals at 0.65 ppm, 0.85 ppm, 0.90 ppm and 0.94 ppm were assigned to the protons of the angular methyl groups of cholesterol (carbons 18, 26 and 27, 21, and 19) [23]. The protons of the sterol skeleton (carbons 3 and 6) in cholesterol were observed at 4.58 ppm and 5.25 ppm. The characteristic peaks of the benzene rings from CH-CHO were observed at 6.63 ppm and 6.84 ppm. Furthermore, the signal for the methine in hydrazone bonds (NH-N=CH) appeared at 8.20 ppm. These results confirmed the successful synthesis of mPEG-CS-Hz-CH.

The degree of substitution (DS) was defined as the number of grafted molecules per 100 sugar units. The DS of mPEG was calculated by measuring the amount of free amino groups using the TNBS method [24]. The calibration curve was constructed using a series of solutions with increasing amounts of CS, and the value was calculated as 9.3%. The DS of CH-CHO was calculated from the integration unit ratio of the NMR peak attributed to the methine group of hydrazone bonds to the characteristic peak of H (b–e) in glucosamine rings, and the value was calculated as 4.0%.

#### 2.2.3. Critical Micelle Concentration (CMC) Determination

**Figure 3:**
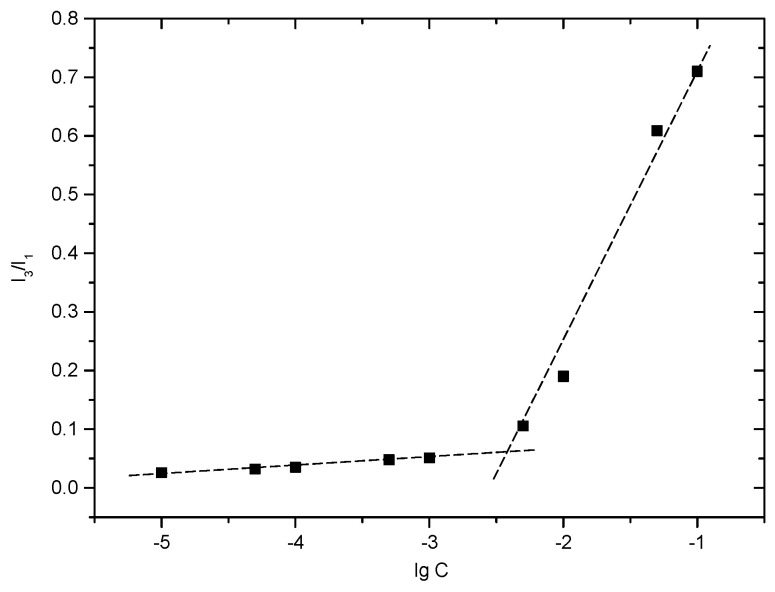
Plot of the fluorescence intensity ratio (I_3_/I_1_) against the logarithm of mPEG-CS-Hz-CH concentration.

CMC is the lowest concentration of amphiphilic polymers needed to form micelles. The self-assembly behavior of mPEG-CH-Hz-CH in the aqueous milieu can be predicted from its CMC value. In this study, the CMC of mPEG-CH-Hz-CH was determined by a fluorescent method. As a hydrophobic fluorescent probe, pyrene is sensitive to the polarity of the environment. With low concentrations of the copolymer, the fluorescent intensity of pyrene remains constant. When the concentration reaches the CMC, micelles are formed spontaneously, and pyrene preferentially resides in the hydrophobic core and emits strongly. The CMC can be determined from the variation in the fluorescence intensity ratio (I_3_/I_1_). As shown in Figure 3, from the cross point of the two straight lines, the CMC of mPEG-CS-Hz-CH was determined as 4.55 × 10^−3^ mg/mL. The low CMC value indicated that the mPEG-CS-Hz-CH micelles would exhibit excellent colloidal stability in dilute conditions, such as in the bloodstream and in bodily fluids.

### 2.3. Preparation of PTX-Loaded mPEG-CS-Hz-CH Micelles

In this study, an ultrasonic probe method was used to prepare the mPEG-CS-Hz-CH micelles. The CH segments formed the hydrophobic core, and PTX could be encapsulated into the core through the hydrophobic interactions. The HA and mPEG segments served as the outer shell, so as to improve the solubility of the drug and enhance the stability of the micelles. The drug loading of mPEG-CS-Hz-CH micelles reached up to 8.74 ± 0.72%, and the encapsulation efficiency was 95.77 ± 3.61%.

### 2.4. Characterization of PTX-Loaded mPEG-CS-Hz-CH Micelles

#### 2.4.1. X-ray Diffraction (XRD) Analysis

**Figure 4:**
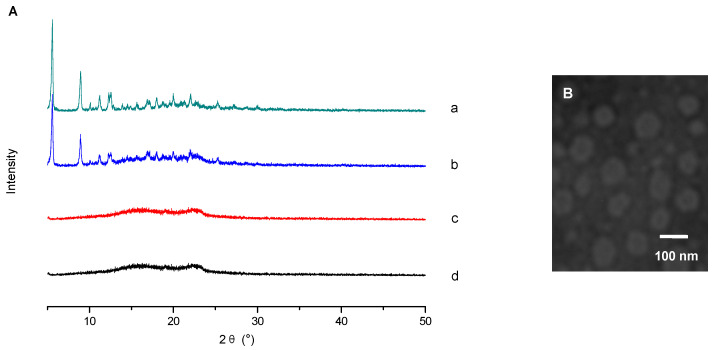
Characterization of PTX-loaded mPEG-CS-Hz-CH micelles. (**A**) XRD spectra of (**a**) PTX, (**b**) physical mixture of PTX and blank mPEG-CS-Hz-CH micelles, (**c**) blank micelles and (**d**) PTX-loaded micelles. (**B**) TEM image of the PTX-loaded mPEG-CS-Hz-CH micelles.

The XRD patterns of PTX, blank mPEG-CS-Hz-CH micelles, their physical mixture and the PTX-loaded micelles are shown in Figure 4A. The diagram of PTX exhibits four intense peaks at 2θ of 5.08°, 8.41°, 10.67° and 11.95°, and there are many small peaks between 15° and 30°. The blank mPEG-CS-Hz-CH micelles showed one “halo”, characteristic of their amorphous state. For their physical mixture, the typical peaks of PTX still existed in the diffractogram. In contrast, the pattern of PTX-loaded mPEG-CS-Hz-CH micelles was similar to that of the blank micelles, which indicated the molecular or amorphous state of PTX in the micelles.

#### 2.4.2. Particle Size and Zeta Potential

The particle size and zeta potential of the mPEG-CS-Hz-CH micelles were determined by the dynamic light scattering (DLS) method. For the blank and PTX-loaded micelles, the mean diameters were 113 ± 2 nm and 146 ± 4 nm, respectively. The difference in their particle size could be due to the encapsulation of the drug. It is well known that particles with a size of no more than 200 nm can passively accumulate in solid tumors via the EPR effect [25]. Moreover, as presented in Figure 4B, the PTX-loaded mPEG-CS-Hz-CH micelles were near spherical in shape and had a narrow distribution.

Zeta potential is a stability predictor of the nano-system. The zeta potential of the blank mPEG-CS-Hz-CH micelles was determined to be +22.4 ± 0.9 mV, and the value was +21.7 ± 0.7 mV for the PTX-loaded micelles. The positive charge was due to the ionized primary amine groups on the glucosamine residues of CS, which could protect the micelles from aggregation by electrostatic repulsion. In addition, it was reported that the surfaces of the tumor cells were negatively charged [26]. The PTX-loaded micelles with a positive charge might have a greater opportunity to bind to the surfaces of tumor cells, which would be beneficial for cellular uptake.

### 2.5. Protein Adsorption

**Figure 5:**
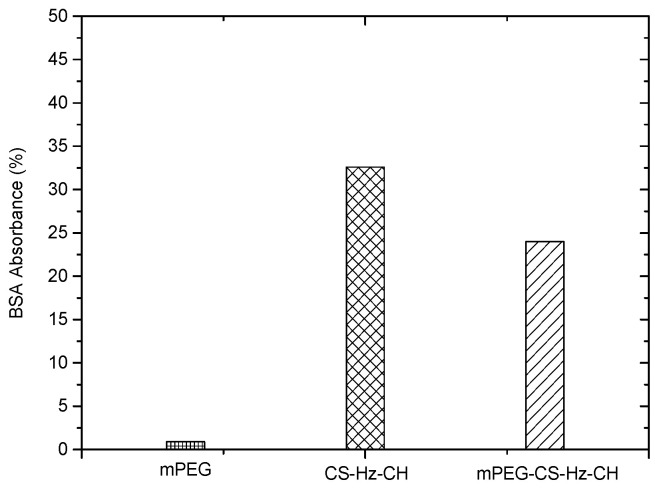
BSA adsorption on mPEG, CS-Hz-CH and mPEG-CS-Hz-CH after incubation at 37 °C for 5 h.

When the drug-loaded micelles enter the bloodstream, the non-specific protein adsorption might trigger a series of undesirable reactions, which would reduce the therapeutic effect of the drug. More specifically, the protein corona would lead to the opsonization of the particles, and the micelles would be easily captured and eliminated by the reticuloendothelial (RES) system. In this study, considering the positive charges on the surfaces of the prepared micelles, negatively charged bovine serum albumin (BSA) was used as a model protein to evaluate the interaction of mPEG-CS-Hz-CH micelles with the protein. As illustrated in Figure 5, CS-Hz-CH displayed significantly high BSA adsorption, with a value of 32.6%. This was mainly due to the strong electrostatic interaction between BSA and CS-Hz-CH. After mPEGylation, the protein adsorption value dramatically decreased to 24.0%, which indicated the significant role of mPEG. Due to its hydrophilic nature and electrical neutrality, mPEG could form a strong hydration layer on the surfaces of the micelles, and the long chain of mPEG could provide high elastic repulsion energy to decrease the interaction between BSA and the particles [27]. It was also reported that the modification by mPEG could protect the micelles from being taken up by the MPS, and a prolonged blood circulation time and enhanced potency of the drug could be obtained [28].

### 2.6. In Vitro pH-Dependent Drug Release Behavior

**Figure 6:**
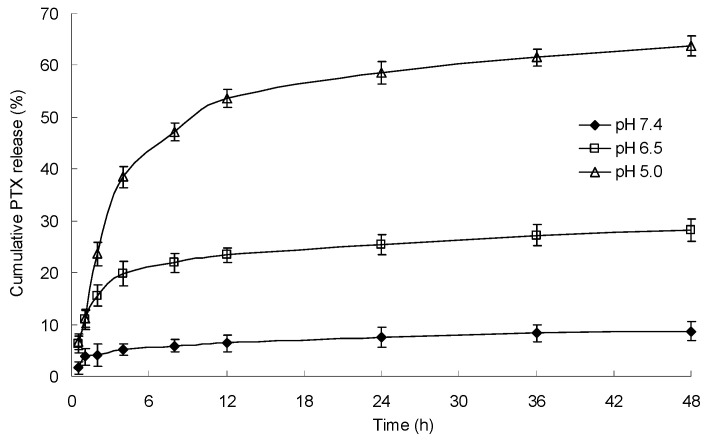
PTX release behavior of the mPEG-CS-Hz-CH micelles under different pH conditions.

The in vitro drug release performance of PTX-loaded mPEG-CS-Hz-CH micelles was investigated under different pH conditions, and the results are presented in Figure 6. In the simulated physiological medium (pH 7.4), the cumulative release percentage of PTX was only 8.7 ± 1.8%, which indicated that the micelles were stable in the normal physiological environment. In comparison, the micelles exhibited an accelerated drug release profile in acidic media, with the cumulative release percentage reaching 28.2 ± 2.1% at pH 6.5 (stimulating the microenvironment of tumor tissue) and 63.8 ± 1.9% at pH 5.0 (stimulating the cytoplasm and endolysosomes of tumor tissue). It was obvious that the drug release behavior of mPEG-CS-Hz-CH micelles was influenced by the pH value, and the lower pH triggered the drug release from the micelles. In an acidic environment, due to the cleavage of hydrazone bonds, the structure of the micelles becomes loose and unstable, thus leading to rapid release of the drug. It could be deduced that the drug release from the micelles might display selectivity in the tumor, which would enhance the drug’s therapeutic efficacy and reduce its toxicity to normal tissue.

### 2.7. In Vitro Cytotoxicity

**Figure 7:**
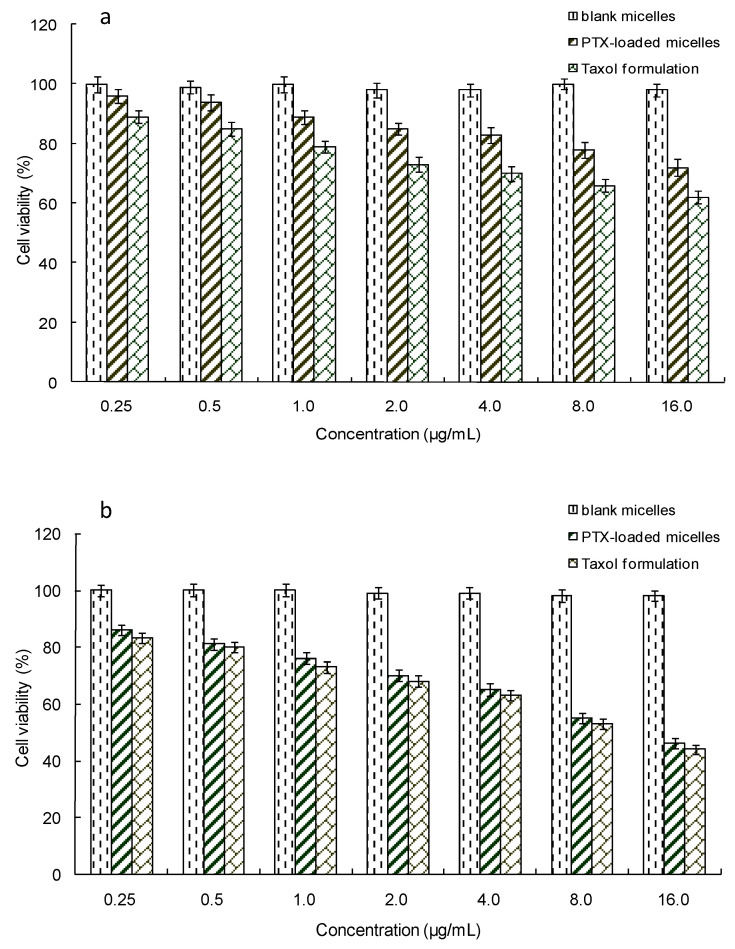
In vitro cytotoxicity of the PTX-loaded mPEG-CS-Hz-CH micelles against (**a**) MCF-10A cells and (**b**) MCF-7 cells.

A standard MTT method was used to evaluate the cytotoxicity of the PTX-loaded mPEG-CS-Hz-CH micelles. As shown in Figure 7, the blank micelles showed negligible cytotoxicity to MCF-7 cells and MCF-10A cells. Even at high concentrations, more than 98% of the cells survived after incubation with the blank micelles. This result demonstrated the non-toxicity of the polymer itself. Moreover, as expected, for PBMC cells, the PTX-loaded micelles exhibited lower cytotoxicity than Taxol, which was due to the pH sensitivity of the micelles. In the cytoplasm of MCF-10A cells, the pH value was 6.8–7.0, and cleavage of the hydrazone bonds in the micelles was not triggered. As a result, the PTX loaded in the micelles displayed a slow release rate, which led to low cytotoxicity. In contrast, for MCF-7 cells, the PTX-loaded micelles exhibited comparable cytotoxicity with Taxol, which could be due to the rapid release of PTX from the micelles. In a weakly acidic tumor intracellular microenvironment, the low pH triggered the cleavage of the hydrazone bonds, and the PTX was released from the micelles quickly. Remarkably, even at the same PTX concentration, the PTX-loaded micelles showed much lower cytotoxicity to normal cells (MCF-10A cells) than to cancer cells (MCF-7 cells). These results implied the significant selectivity of PTX-loaded mPEG-CS-Hz-CH micelles on tumor cells. It could be concluded that the mPEG-CS-Hz-CH micelles might be an ideal nanocarrier system for the delivery of PTX.

### 2.8. In Vivo Antitumor Efficacy

**Figure 8:**
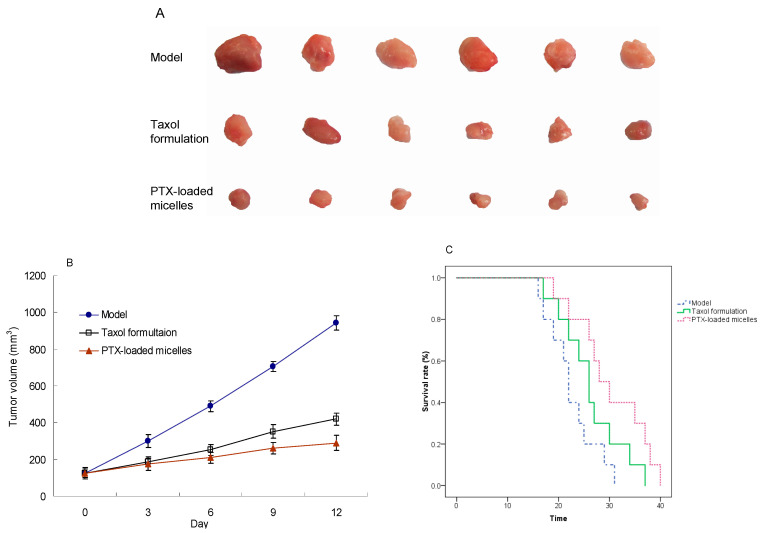
In vivo antitumor effect of the PTX-loaded mPEG-CS-Hz-CH micelles. (**A**) Tumors excised from the mice after different intravenous injection treatments; (**B**) Variation in tumor volume after treatment; (**C**) Kaplan–Meier survival curves of the tumor-bearing mice after treatment.

The antitumor activity of the PTX-loaded mPEG-CS-Hz-CH micelles was further investigated in tumor-bearing mice. The tumor volumes of each group were measured and plotted as a function of time. As shown in Figure 8A, tumors in the normal saline group grew rapidly. In comparison, both PTX formulations showed efficient tumor inhibition effects, and the PTX-loaded micelles group displayed much smaller tumors than the Taxol group, with a TIR of 71.3% vs. 59.4%.

Moreover, as a gold standard for the evaluation of antitumor drugs, the survival time of tumor-bearing mice in each group after the treatment was determined. As shown in Figure 8B, all of the normal saline-treated mice died within 31 days, which was attributed to the rapid growth of the tumor and tumor-associated complications. In comparison, the PTX-loaded micelles and Taxol formulation significantly prolonged the survival time of the tumor-bearing mice. At day 25, the survival rates of the model group, Taxol group and PTX-loaded mPEG-CS-Hz-CH micelles group were 30%, 60% and 80%, respectively. The median survival time was 22 days, 26 days and 28 days, respectively, which implied the superior antitumor efficacy and safety of the PTX-loaded mPEG-CS-Hz-CH micelles.

The remarkable antitumor efficacy of the PTX-loaded micelles could be explained by the following factors. First, the hydrophilic outer layers formed by the mPEG segments could reduce the non-specific protein adsorption of the PTX-loaded mPEG-CS-Hz-CH micelles, so that the micelles could escape immune clearance by the RES and prolong the circulation time of the drug. With a size in the range of 100–200 nm, the micelles could have a greater opportunity to accumulate in the tumor via the enhanced EPR effect. Second, it was easy for the positively charged PTX-loaded micelles to attach to the surfaces of the negatively charged tumor cells, which facilitated the cellular uptake. Moreover, the hydrazone bonds in the micelles led to specific and efficient release of the drug at the tumor site, which could enhance the antitumor efficacy and reduce the toxicity to normal tissue. In summary, the mPEG-CS-Hz-CH micelles would be ideal nanocarriers for the delivery of anticancer drugs.

## 3. Materials and Methods

### 3.1. Materials

Chitosan (Mw = 30 kDa, more than 97% deacetylated) was purchased from Kittolife Co., Ltd., Seoul, Korea. Poly(ethylene glycol) methyl ether (mPEG, Mw = 1900 Da), 4-formylbenzoic acid, cholesterol (CH), monoethyl succinate, hydrazine monohydrate, N-hydroxysuccinimide (NHS) and 1-(3-dimethylaminopropyl)-3-ethylcarbodiimide hydrochloride (EDC) were purchased from Aladdin Industrial Co., Shanghai, China. Pyrene and 3-(4,5-dimethylthiazol-2-yl)-2,5-diphenyl tetrazolium bromide (MTT) were purchased from Sigma-Aldrich Co., St. Louis, MO, USA. Paclitaxel (PTX, purity of 99.9%) was obtained from Natural Field Biological Technology Co., Ltd., Xi’an, China. Dulbecco’s modified Eagle’s medium (DMEM), bovine serum albumin (BSA), fetal bovine serum (FBS) and phosphate-buffered saline (PBS) were purchased from Gibco BRL, Carlsbad, CA, USA. All other reagents were of analytical or chromatographic grade and used without further purification.

### 3.2. Cell Lines and Animals

The PBMC cells (peripheral blood mononuclear cells, normal cells), MCF-7 cells (human breast cancer cells), MCF-10A cells (healthy human breast epithelial cells) and H22 cells (mouse hepatocellular carcinoma cells) were obtained from Harbin Medical University. Cells were cultured in DMEM containing 10% FBS and 1% penicillin–streptomycin at 37 °C in an atmosphere of 5% CO_2_ and 95% humidified air.

Specific pathogen-free Kunming mice (20–25 g) were supplied by the Laboratory Animal Center of Harbin Medical University. Animals were fed under conditions of 25 °C and 55% humidity. All animal studies were performed in compliance with the guidelines of the Animal Ethics Committee of Heilongjiang University.

### 3.3. Synthesis of mPEG-CS-Hz-CH

The copolymer mPEG-CS-Hz-CH was prepared by reactions involving several steps. First, 4-formylbenzoic acid was conjugated with cholesterol to form CH-CHO. Next, for CS, the modification by mPEG was performed to obtain mPEG-CS. Then, the mPEG-CS was functionalized with monoethyl succinate to obtain mPEG-CS-COOC_2_H_5_. After further reaction with hydrazine monohydrate, the mPEG-CS-NH-NH_2_ was obtained. Finally, the final product of mPEG-CS-Hz-CH was synthesized via the reaction between CH-CHO and mPEG-CS-NH-NH_2_.

#### 3.3.1. Synthesis of CH-CHO

CH-CHO was synthesized by the coupling reaction between carboxyl groups of 4-formylbenzoic acid and hydroxyl groups of cholesterol in the presence of EDC and NHS. Briefly, 150 mg of 4-formylbenzoic acid and 386 mg of cholesterol were dissolved in 20 mL of DMF, with the addition of 287 mg of EDC and 173 mg of NHS to activate the carboxyl groups. The reaction was allowed to proceed for 72 h under stirring at 45 °C. DMF was removed under reduced pressure at 65 °C. Then, 10 mL of distilled water was added into the resultant product, and the mixture was stirred for 12 h at room temperature to remove EDC and NHS. After filtration and drying, the product CH-CHO was obtained.

#### 3.3.2. Synthesis of mPEG-CS-NH-NH_2_

Firstly, CS was modified with mPEG as follows: 120 mg of CS and 480 mg of mPEG were dissolved in 24 mL of 17% acetic acid solution, and the mixture was stirred for 15 min at room temperature. After addition of 21 mL of 37% formaldehyde, vigorous stirring was continued for a further 1 h, and the resultant product was dialyzed (MWCO: 30 kDa) against distilled water for 48 h in order to remove the unreacted chemicals. The mPEG-CS powder was obtained after lyophilization.

Secondly, mPEG-CS was functionalized with monoethyl succinate to obtain mPEG-CS-COOC_2_H_5_. Briefly, 5.4 mg of monoethyl succinate, 11 mg of EDC, 6 mg of NHS and 150 mg of mPEG-CS were dissolved in 20 mL of distilled water, and the mixture was stirred at 40 °C for 24 h. After the reaction was completed, the resultant product was dialyzed (MWCO: 14 kDa) against distilled water for 24 h for purification. The mPEG-CS-COOC_2_H_5_ was obtained by lyophilization.

Thirdly, mPEG-CS-COOC_2_H_5_ was reacted with hydrazine monohydrate to form mPEG-CS-NH-NH_2_. In brief, 150 mg of mPEG-CS-COOC_2_H_5_ was dissolved in 15 mL of distilled water, and then 0.5 mL of hydrazine monohydrate was added dropwise. The reaction was allowed to proceed at 37 °C for 2 h. The resultant product was purified by dialysis against distilled water for 24 h (MWCO: 14 kDa), and the product was isolated by lyophilization.

#### 3.3.3. Synthesis of mPEG-CS-Hz-CH

The polymer mPEG-CS-Hz-CH was prepared by covalently conjugating mPEG-CS-NH-NH_2_ with CH-CHO. As is typical, 140 mg of mPEG-CS-NH-NH_2_ and 130 mg of CH-CHO were dissolved in 5 mL of DMF. The mixture was stirred at 37 °C for 12 h. At a predetermined time, the resultant product was dialyzed against distilled water (MWCO: 14 kDa) for 24 h and then lyophilized to obtain the mPEG-CS-Hz-CH powder.

### 3.4. Characterization of mPEG-CS-Hz-CH

To confirm the successful synthesis of mPEG-CS-Hz-CH, FT-IR analysis and ^1^H NMR analysis were performed. The CMC of this amphiphilic polymer was determined using pyrene as the fluorescence probe [29].

### 3.5. Preparation and Characterization of PTX-Loaded mPEG-CS-Hz-CH Micelles

The incorporation of PTX into the mPEG-CS-Hz-CH micelles was achieved by a sonication method. As is typical, a certain amount of mPEG-CS-Hz-CH was dispersed in deionized water (0.25 mg/mL). Afterwards, 1 mL of PTX–acetone solution (0.25 mg/mL) was added, and the mixture was sonicated at 400 W for 6 min (pulse on 2 s and pulse off 1 s). During this process, the sample was kept in an ice cooling bath to prevent overheating. After ultrasonication, the mixture was dialyzed against distilled water (MWCO: 14 kDa) for 3 h to remove acetone and the unloaded PTX. Finally, the resultant product was lyophilized to obtain the PTX-loaded mPEG-CS-Hz-CH micelle powder. The blank micelles were prepared by the same process without addition of PTX.

For characterization, X-ray diffraction analysis was performed (Geigerflex, Rigaku Co., Tokyo, Japan). The DLS technique was used to measure the particle size and zeta potential of the PTX-loaded mPEG-CS-Hz-CH micelles (Malvern Instruments Zetasizer Nano-ZS90, Malvern, UK). The morphology of the PTX-loaded micelles was observed by transmission electron microscopy (TEM, HT7700, Hitachi Ltd., Tokyo, Japan). The drug encapsulation efficiency (*EE%*) and drug loading (*DL%*) were determined by an HPLC system at 227 nm, and the following equations were used for calculation [29].

*EE*% = mass of PTX in micelles/mass of PTX added during preparation × 100% (1)

*DL*% = mass of PTX in micelles/mass of polymeric micelles × 100% (2)

### 3.6. Protein Adsorption Test

The stability of the mPEG-CS-Hz-CH micelles in the bloodstream was evaluated by a protein adsorption test. BSA was used as a model protein. A series of BSA in PBS 7.4 solutions with concentrations from 0.1 mg/mL to 0.7 mg/mL were tested by a fluorescence spectrophotometer (RF-6000, Shimadzu, Tokyo, Japan) with the excitation wavelength of 285 nm. The absorbance values determined at 337 nm were plotted against BSA concentrations to obtain the standard curve. Then, mPEG, CS-Hz-CH and mPEG-CS-Hz-CH were incubated with the BSA solution, respectively, with the final polymer concentration of 0.25 mg/mL, and the BSA concentration of 0.5 mg/mL. After incubation at 37 °C for 5 h, samples were centrifuged at 18,000 rpm for 15 min. The supernatant was analyzed to determine the amount of adsorbed BSA.

### 3.7. In Vitro Release Study

To investigate the potential application of mPEG-CS-Hz-CH micelles as pH-sensitive drug delivery vehicles, the in vitro release study was performed by a dialysis method. Two milliliters of PTX-loaded micelles were put into a dialysis bag (MWCO: 13.5 kDa), which was then placed in 50 mL of release medium. PBS solutions containing 0.1% (*w/v*) Tween 80 with different pH values (5.0, 6.5 and 7.4) were used as the media. At appropriate time intervals, samples were withdrawn from the exterior solution and equal volume of fresh medium was added back to keep the total volume constant. The percentage of PTX released was estimated by an HPLC method.

### 3.8. In Vitro Cytotoxicity

The in vitro cytotoxicity of PTX-loaded mPEG-CS-Hz-CH micelles against MCF-7 cells was assessed by MTT assay using MCF-10A cells for comparison. Cells in the logarithmic growth phase were seeded in 96-well plates (approximately 1 × 10^4^ cells per well). When cell confluence reached 70–80%, different concentrations of samples were added. After 24 h of incubation, wells were rinsed with PBS and then 10 μL of MTT solution (5 mg/mL) was added to each well. The cells were further incubated for 4 h. At a predetermined time point, the unreacted MTT was aspirated off and the formazan crystals were dissolved with 100 μL of DMSO. The absorbance at 490 nm was determined by a BioRad microplate reader (Bio-Rad 680, Bio-Rad Laboratories, Hercules, CA, USA). Cells without any treatment were used as the control. To evaluate the effect of samples on the cell proliferation, cell viability was calculated as follows:

Cell viability (%) = A_sample_/A_control_ × 100% (3)

where A_sample_ was the absorbance value of cells treated with the sample, and A_control_ was the absorbance of untreated cells.

### 3.9. In Vivo Antitumor Efficacy Study

H22 tumor-bearing mice were used to further evaluate the in vivo antitumor activity of the PTX-loaded mPEG-CS-Hz-CH micelles. H22 transplanted mouse models were established via the subcutaneous injection of 100 μL of H22 cell suspension (containing 2 × 10^6^ cells) into the right flank of each mouse. When the tumor became palpable (set as day 0), the mice were weighed and randomly divided into three groups (*n* = 6), which were treated with normal saline, Taxol (PTX of 15 mg/kg) and PTX-loaded micelles (PTX of 15 mg/kg). Samples were administrated through tail vein injection on days 0, 3, 6 and 9. The tumor size was monitored every 3 days to estimate the tumor volume via Equation (4). At the end of the experiment (day 12), the mice were sacrificed. The tumor tissues were excised, measured, weighed and photographed. The tumor inhibition rate (TIR) was calculated to evaluate the antitumor efficacy of the PTX-loaded micelles quantitatively.

Tumor volume = 1/2 × a × b^2^ (4)

where a and b represented the longest diameter and the shortest diameter of the tumor, respectively.

TIR (%) = (W_model_ − W_treated_)/W_model_ × 100% (5)

where W_model_ was the average weight of tumor tissues from the normal saline group, and W_treated_ was the average weight of tumor tissues from the treated group.

Additionally, a similar process was performed to determine the survival time of the mice in each group (*n* = 10). After injections, the mice were allowed unrestricted access to food and water until death, and the Kaplan–Meier survival curves of each group were plotted.

### 3.10. Statistical Analysis

All experimental data were expressed as the average value ± standard deviation (SD). Statistical comparison was conducted using Student’s *t*-test, and the results were considered statistically significant when *p* < 0.05.

## 4. Conclusions

In this study, an amphiphilic polymer of mPEG-CS-Hz-CH was successfully synthesized. The polymer could self-assemble into micelles in aqueous milieu and encapsulate the PTX with high drug loading. The mPEG-CS-Hz-CH micelles displayed a pH-dependent drug release profile. The in vitro cytotoxicity study demonstrated the non-toxicity of the polymer itself, and the PTX-loaded micelles exhibited significant selectivity on tumor cells. Moreover, the PTX-loaded mPEG-CS-Hz-CH micelles exhibited comparable cytotoxicity with Taxol against MCF-7 cells. The in vivo antitumor efficacy study confirmed that the PTX-loaded micelles could improve the therapeutic efficacy of PTX and reduce the side effects. It can be concluded that the mPEG-CS-Hz-CH micelles might be a promising pH-sensitive drug delivery carrier for anticancer drugs.

## Data Availability

Data is contained within the article.
